# Compact and highly-confined spoof surface plasmon polaritons with fence-shaped grooves

**DOI:** 10.1038/s41598-019-48616-0

**Published:** 2019-08-19

**Authors:** Richeng Ping, Hong Ma, Yang Cai

**Affiliations:** Department of Electronic and Optical Engineering, Space Engineering University, Beijing, 101400 China

**Keywords:** Electronic devices, Nanophotonics and plasmonics

## Abstract

In this paper, a compact and highly-confined spoof surface plasmon polaritons (SSPPs) with fence-shaped grooves is proposed. By adding the metal strips that similar to the fence on the basis of T-grooves, the waves can be confined more tightly as the propagation paths of current are effectively increased, which leads to a reduction of the height of SSPPs units by 48.4% compared to the rectangular grooves. Moreover, compared with conventional SSPPs units mentioned before, the proposed design exhibits the strongest confinement to EM waves. To verify the effectiveness of the proposed unit, a corresponding transmission line is simulated and fabricated. Both simulated and measured results are in good agreement, which show the superior performance of this structure. Meanwhile, due to the simplicity and effectiveness, this proposed structure has a great research and application prospects in developing the miniaturized devices and circuits.

## Introduction

Surface plasmon polaritons (SPPs) are surface electromagnetic (EM) waves that transmit along the interface between metals and dielectrics because of the coupling between free electrons and photons^1–4^. Compared with the wave vector of photons in dielectrics, the propagation constant of SPPs is much larger which makes it possesses the ability to break the traditional diffraction limit and are expected to be used in new generation of miniaturized photonic devices and circuits^5–7^. However, natural SPPs at terahertz (THz) and microwave bands are not available since metals behave like perfect electric conductors (PECs) at these frequencies^8–12^. Recently, spoof surface plasmon polaritons (SSPPs) are proposed by Pendry and his co-workers, which are realized by drilling periodic arrays of holes or grooves so that metals can support surface waves which is similar to optical SPPs in THz and microwave bands^13,14^. Furthermore, the proposal of planar SSPPs that perfectly solves the problem of the massive volume of the three-dimensional structures extremely promotes the research about the characteristics of SSPPs^15,16^.

A remarkable advantage of SSPPs is that the dispersion relations and field confinement can be directly manipulated by changing its structural parameters. Hence, various SSPPs units, including rectangular grooves^17^, V-grooves^18^, dumbbell grooves^19^, trapezoidal grooves^20^, T-grooves^21^ and parallel–arranged grooves^22^ are designed and simulated to improve the integration of the circuits.

In this paper, a novel SSPPs with fence-shaped grooves is proposed which can be implemented by adding the metal strips that similar to the fence on the basis of T-grooves. Compared with other kinds of SSPPs units, the structure mentioned here possesses the strongest confinement of EM waves and greatly reduce the size under the same cut-off frequency. A transmission line based on the proposed structure is simulated and fabricated, both simulated and measured results are in good agreement which shows the excellent properties of this structure.

## Results

### Dispersion relations of plasmonic waveguides with different SSPPs units

**Figure 1:**
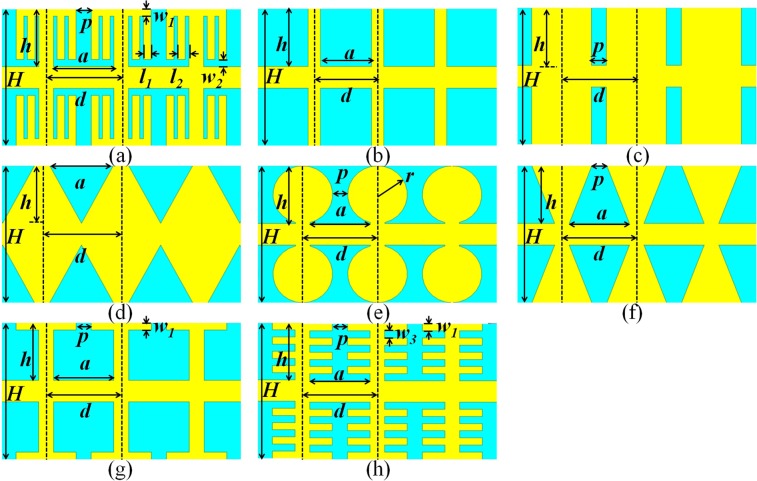
(**a**) Fence-shaped grooves. (**b**) Rectangular grooves with slot width of a. (**c**) Rectangular grooves with slot width of p. (**d**) V-grooves. (**e**) Dumbbell grooves. (**f**) Trapezoidal grooves. (**g**) T-grooves. (**h**) Parallel-arranged grooves.

The schematic configuration of the proposed plasmonic periodic structure with fence-shaped grooves is shown in Fig. 1(a), which is obtained by loading the fence-shaped metal strips of the same size on both sides of SSPPs units with T-grooves. This structure is divided into two layers, the yellow part represents the copper with a thickness of 0.018 *mm*, while the blue part is the substrate of Rogers 5880 whose thickness and dielectric constant are 0.508 *mm* and 2.2, respectively. The height of the metal strip and the grooves are set as *H* and *h*, the top and bottom lengths of grooves are expressed as *p* and *a*, the periodic length is marked as *d*. Meanwhile, the width of the top of the T-grooves, the distance between the metal strips and the T-grooves, the width of the metal strips and its periodic are denoted as *w*_1_, *w*_2_, *l*_1_ and *l*_2_, respectively. In contrast, the rest units introduced as shown in Fig. 1(b–h) are also two-layer structures, which are rectangular grooves, V-grooves, dumbbell grooves, trapezoidal grooves, T-grooves and parallel-arranged grooves. All the parameters can be adjusted according to the actual model to obtain the different degrees of limitations on EM waves.

**Figure 2:**
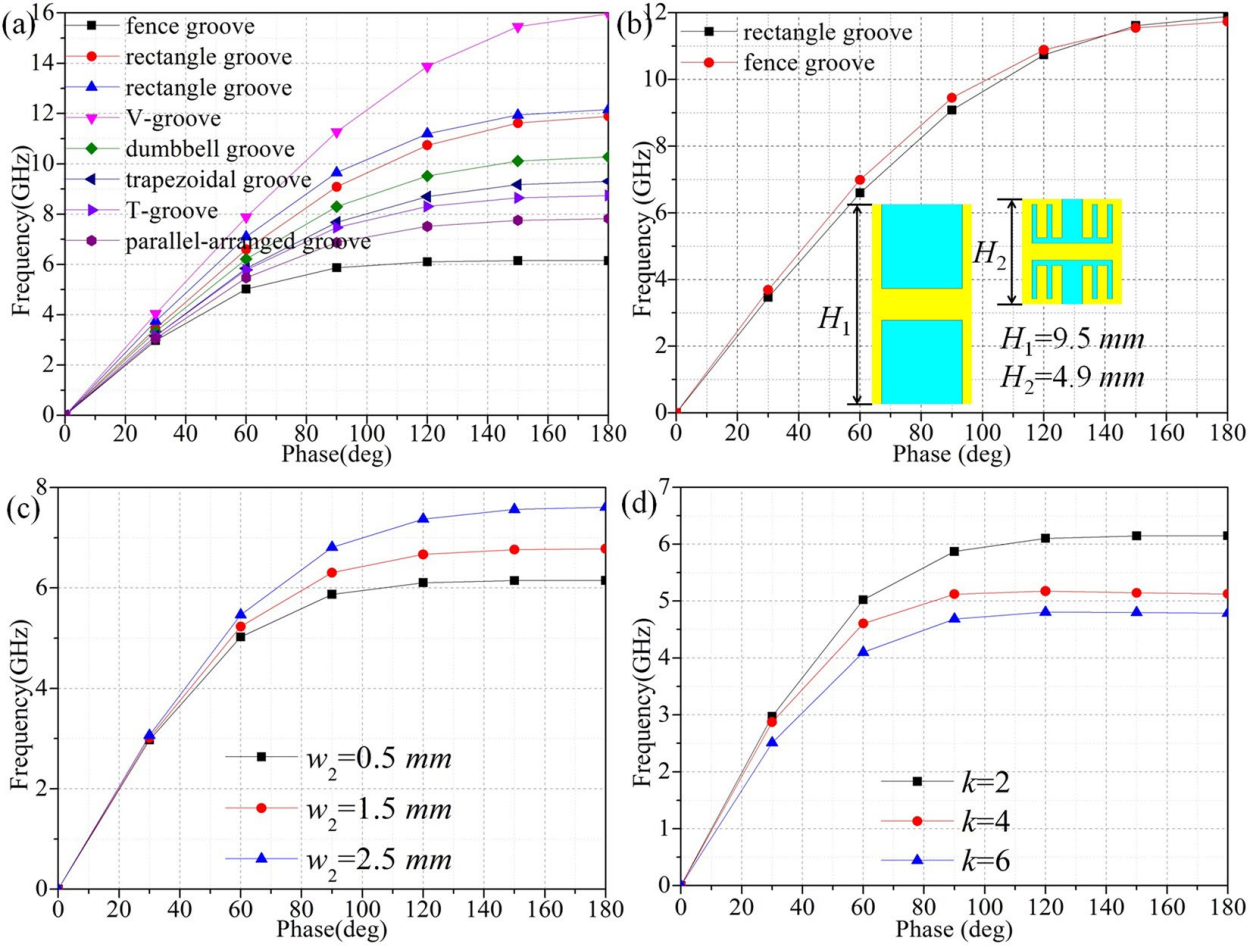
(**a**) The dispersion relations of different SSPPs units. (**b**) The dispersion curves possess the same cut-off frequency of rectangular grooves and fence-shaped grooves with heights *H*_1_ = 9.5 *mm* and *H*_2_ = 4.9 *mm*, respectively. (**c**) The dispersion curves of fence-shaped grooves with different *w*_2_. (**d**) The dispersion curves of fence-shaped grooves with different *k*.

In order to compare the limitation of EM waves of different structures shown in Fig. 1. The above parameters and *w*_3_ appears in Fig. 1(h) are set as *H* = 9.5 *mm*, *h* = 4 *mm*, *p* = 1 *mm*, *a* = 4 *mm*, *d* = 5 *mm*, *w*_1_ = 0.5 *mm*, *w*_2_ = 0.5 *mm*, *l*_1_ = 0.5 *mm*, *l*_2_ = 0.75 *mm*, *w*_3_ = 0.5 *mm*, in which the variables can be controlled effectively and make the results more convincing. The commercial simulation software CST is used to analyze the dispersion characteristics of these units. It can be clearly seen in Fig. 2(a) that the cut-off frequency of V-grooves is the biggest, which is 15.96 GHz, followed by rectangle grooves(12.15 GHz, 11.88 GHz), dumbbell grooves(10.28 GHz), trapezoidal grooves(9.3 GHz), T-grooves(8.74 GHz), parallel-arranged grooves(7.82 GHz) and fence-shaped grooves(6.14 GHz), which is the smallest. It indicates that the proposed unit has the strongest confinement to EM waves. Simultaneously, to investigate the influence of different parameters on the dispersion relations of the proposed unit, the number of fence-shaped metal strips loaded on T-grooves, which is assigned as *k* equals to 2, 4 and 6. Moreover, the *w*_2_ is given as 0.5 *mm*, 1.5 *mm* and 2.5 *mm* respectively to get their dispersion curves.

The simulated results of the above cases are shown in Fig. 2, from which it can be found that whether the number of metal strips is more or *w*_2_ is smaller, the factor that changes with them is only the current path which is marked by letter *l* becomes longer and results show that the cut-off frequency decreases. Therefore, it can be concluded that the cut-off frequency is not only determined by the groove depth *h*, but also by the length of the current path *l*. Moreover, the fence-shaped grooves can reach a decrease of 48.4% in height compared with rectangular grooves which will benefit the miniaturization of circuits definitely.

**Figure 3:**
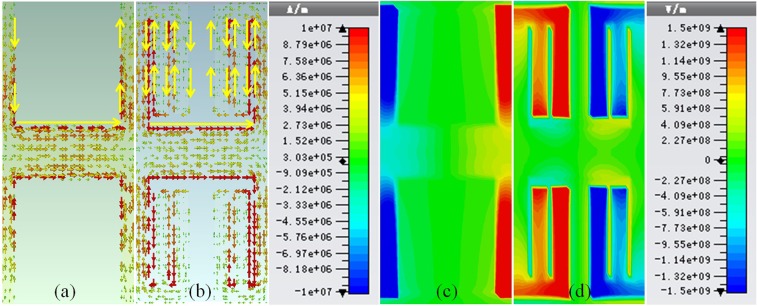
(**a**) The current distributions of rectangular grooves. (**b**) The current distributions of fence-shaped grooves. (**c**) The simulated confinement of EM waves in rectangular grooves. (**d**) The simulated confinement of EM waves in fence-shaped grooves.

The current distributions of the proposed grooves and rectangular grooves are simulated and shown in Fig. 3(a,b). It can be clearly seen that the propagation path of the current in fence-shaped grooves is much longer than that in rectangular grooves, which proves the previous inference.

Meanwhile, as shown in Fig. 3(c,d), the confinement of EM waves is much more significant in fence-shaped grooves than that in rectangular grooves.

### The simulated and measured results of the proposed SSPPs waveguides with fence-shaped grooves

**Figure 4:**
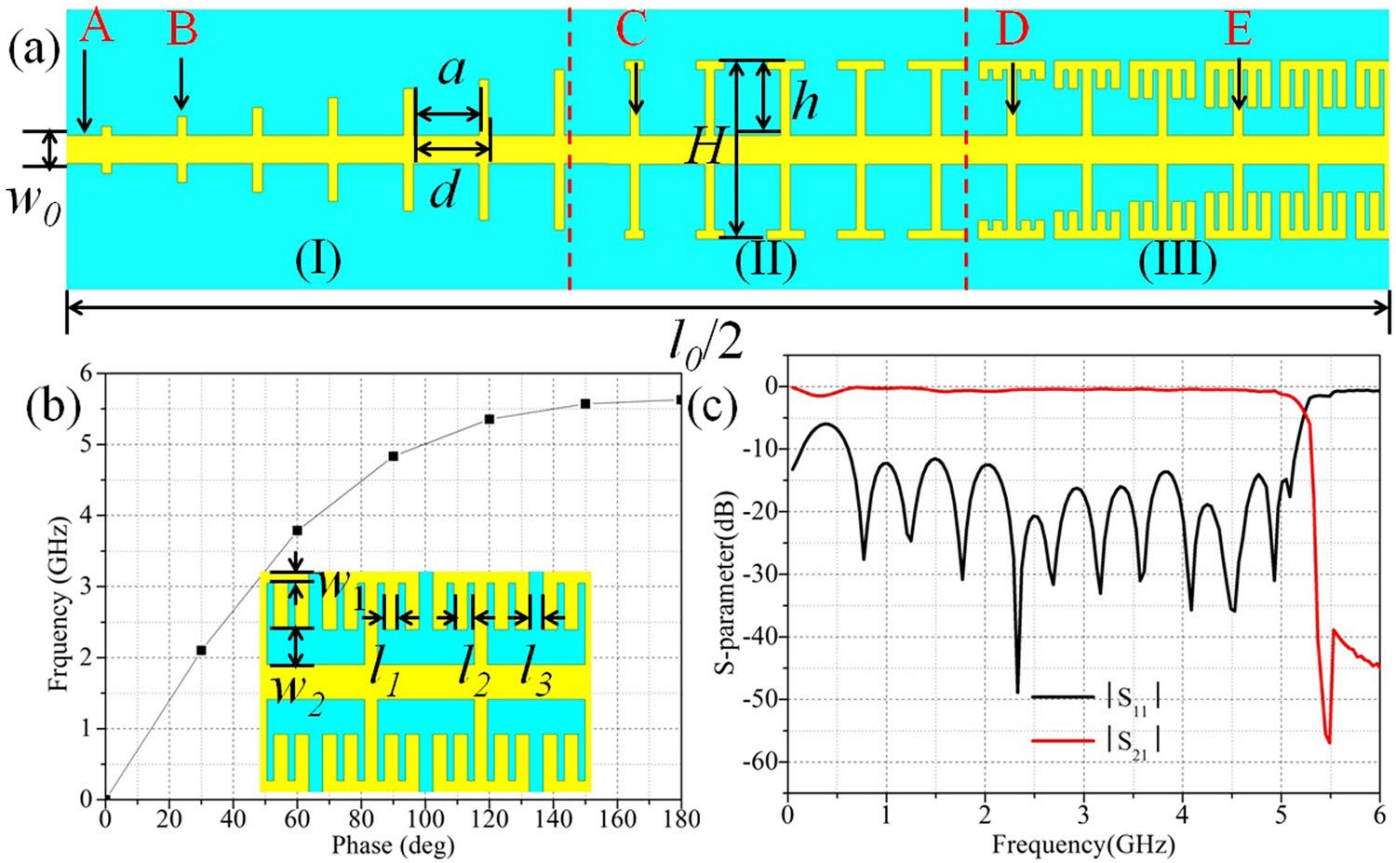
(**a**) The schematic diagram of the transmission line. (**b**) The dispersion relation of the fence-shaped SSPPs unit with three layers. (**c**) The S parameter curves of the transmission line.

**Figure 5:**
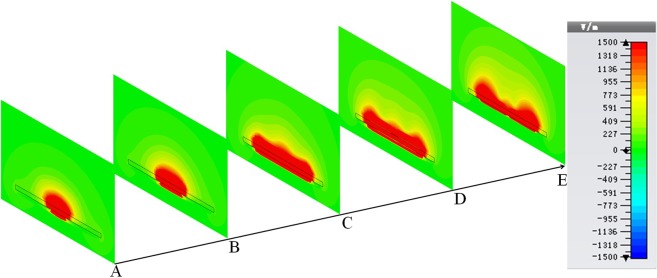
The simulated electric-field distributions of five different cross sections (**A**–**E**).

Based on the proposed structure, a transmission line shown in Fig. 4(a) is designed which completes the transition of rectangular grooves, T-grooves and finally, the fence-shaped grooves that displayed in part (I), (II) and (III), respectively. As the microstrip feeding structure is adopted here, this line is divided into three layers, the upper and lower layers are copper sheets with the thickness of 0.018 mm that are used to fabricate the transmission part and ground plane, and the middle layer is dielectric substrate made of Rogers 5880, whose thickness and dielectric constant are 0.508 mm and 2.2, respectively. Meanwhile, due to the three-layer structure the transmission line is, the dispersion curve of the SSPPs units on the line is re-simulated which shows the cut-off frequency is about 5.6 GHz and the units are also three-layer structures. The height of the metal strip and groove are set as *H* = 9.5 *mm* and *h* = 4 *mm* to match the impedance of 50 ohm. The other parameters in Fig. 4 are marked as *w*_0_ = 1.5 *mm*, *w*_1_ = 0.5 *mm*, *w*_2_ = 1.5 *mm*, *l*_0_ = 140.5 *mm*, *l*_1_ = 0.5 *mm*, *l*_2_ = 0.75 *mm*, *a* = 3.5 *mm*, *d* = 4 *mm*. The S-parameter diagrams shown in Fig. 4(c) indicates that the cut-off frequency of the transmission line is about 5.4 GHz, which is in good agreement with that of the units. Besides, the results show the excellent performance of this structure. Figure 5 shows the electric-field distributions in five different cross sections A, B, C, D, E that marked in Fig. 4, which not only clarifies that the electric field is restricted under the SSPPs units tightly, but also demonstrates the gradual transition of the field from microstrip line to SSPPs clearly.

**Figure 6:**
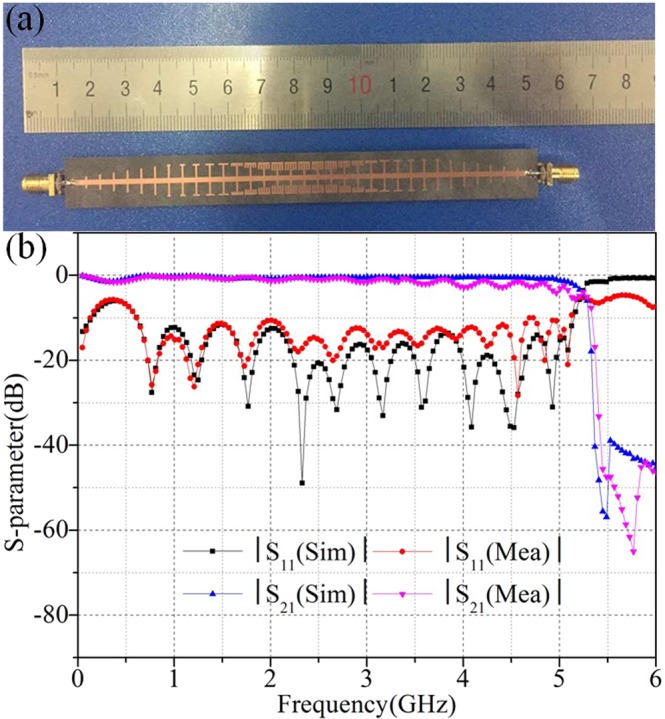
(**a**) The photograph of the fabricated proposed transmission line. (**b**) The simulated and measured |S_11_| and |S_21_| of the fabricated sample.

Furthermore, to verify the functionality of the SSPPs with fence-shaped grooves, a device shown in Fig. 6(a) is made and tested according to the size and materials of the transmission line. The S-parameter of this line is measured by Vector Network Analyzer (N5247A) and the simulated and measured results are compared in Fig. 6(b). Due to the existence of machining error, testing errors and errors caused by human factors, all of these will lead to the difference between simulated and measured results. Despite these errors, it is clear from the Fig. 6(b) that the two results are in good agreement and |S_11_| is less −10 dB from 0.6 GHz to 5 GHz, while |S_21_| is higher than −1dB from 0.5 GHz to 4 GHz, which not only indicate the efficient transmission of this line, but also prove the validity of the proposed structure.

## Conclusions

In summary, a novel spoof surface plasmon polaritons with fence-shaped grooves of compact size is proposed in this paper. For the propagation path of current is effectively increased, the fence-shaped grooves possess the strongest confinement to EM waves compared with other SSPPs units, which can be well utilized in the size reduction. For validation, a transmission line based on it is simulated and fabricated, the results of both are in good agreement and show the great performance of this structure. Due to the simplicity and maneuverability the fence-shaped SSPPs unit has, it is expected that this structure will be very promising in realizing the compact and integrated plasmonic devices and circuits.

## Methods

All numerical simulations including dispersion relations, current and E-field distributions and S parameters are conducted by using the eigen-mode solver and time-domin solver of the commercial software, CST Microwave Studio. The experimental structure is fabricated by using a 0.508 mm thickness dielectric film, Rogers 5880, whose relative permittivity and tangent loss are 2.2 and 0.0009, respectively. The thickness of copper film is 0.018 mm. In the experiment, the Agilent Vector Network Analyzer (N5247A) is used to measure the S parameters, including the reflection coefficients |S_11_| and transmission coefficients |S_21_| of the fabricated sample.

## Data Availability

All data generated or analysed during this study are included in this published article (and its Supplementary Information files).
